# Assessing fluid status with the vascular pedicle width: relationship to IVC diameter, IVC variability and lung comets

**DOI:** 10.1186/cc14267

**Published:** 2015-03-16

**Authors:** N Salahuddin, I Hussain, Q Shaikh, M Joseph, H Alsaidi, K Maghrabi

**Affiliations:** 1 King Faisal Specialist Hospital & Research Centre, Riyadh, Saudi Arabia

## Introduction

This study attempts to determine a vascular pedicle width (VPW) cutoff value that identifies a fluid replete state defined as an IVC diameter ≥2 cm and ≤15% respiratory variation.

## Methods

In a cross-sectional design, consecutive, critically ill patients underwent simultaneous chest radiographs and ultrasounds. The Research Ethics Committee approved the study.

## Results

**Figure 1:**
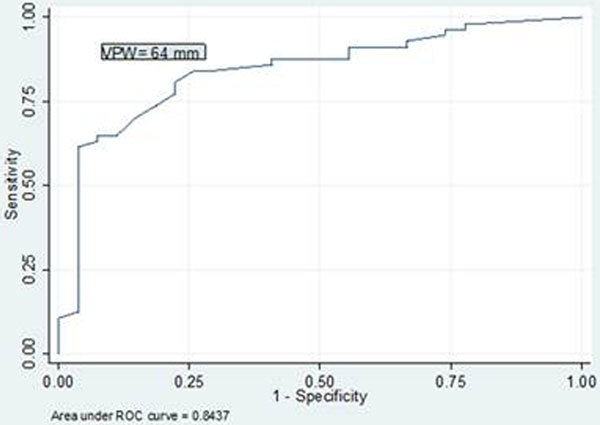
**ROC curve for VPW discriminating fluid repletion by IVC ultrasound**.

Eighty-four data points on 43 patients were collected. VPW correlated with IVC diameter (*r *= 0.64, *P *≤0.001) and IVC variation (*r *= -0.55, *P *≤0.001). No correlation was observed between VPW and number of lung comets (*r *= 0.12, *P *= 0.26) or positive fluid balance (*r *= 0.3, *P *= 0.058). On multivariate linear regression, standardized coefficients demonstrated that a 1 mm increase in IVC diameter corresponded to a 0.28 mm (Beta) increase in VPW. ROC curve analysis yielded an AUC of 0.843 (95% CI = 0.75 to 0.93), *P *≤0.001 and provided the best accuracy with a cutoff VPW value of 64 mm (sensitivity 81%, specificity 78%, PPV = 88.5%, NPV = 66%, correct classification rate = 79.6%). See Figure 1.

## Conclusion

A VPW value of 64 mm accurately identifies a fluid replete state. Increased extravascular lung water, however, was not relatable to the VPW measurements. The VPW can be confidently used to discriminate fluid repletion from fluid responsiveness.

